# Supplementary material for: High-throughput evaluation of genetic variants with prime editing sensor libraries
Source: Nat Biotechnol. 2024 Mar 12;43(10):1648–62. doi: 10.1038/s41587-024-02172-9 (PMC12520993; doi:10.1038/s41587-024-02172-9)
Supplement: Supplementary file 1 — Supplementary Fig. 1 and Protocols 1–3. [file 41587_2024_2172_MOESM1_ESM.pdf]

nature biotechnology

Article https://doi.org/10.1038/s41587-024-02172-9

High-throughput evaluation of genetic variants with prime editing sensor libraries

In the format provided by the authors and unedited

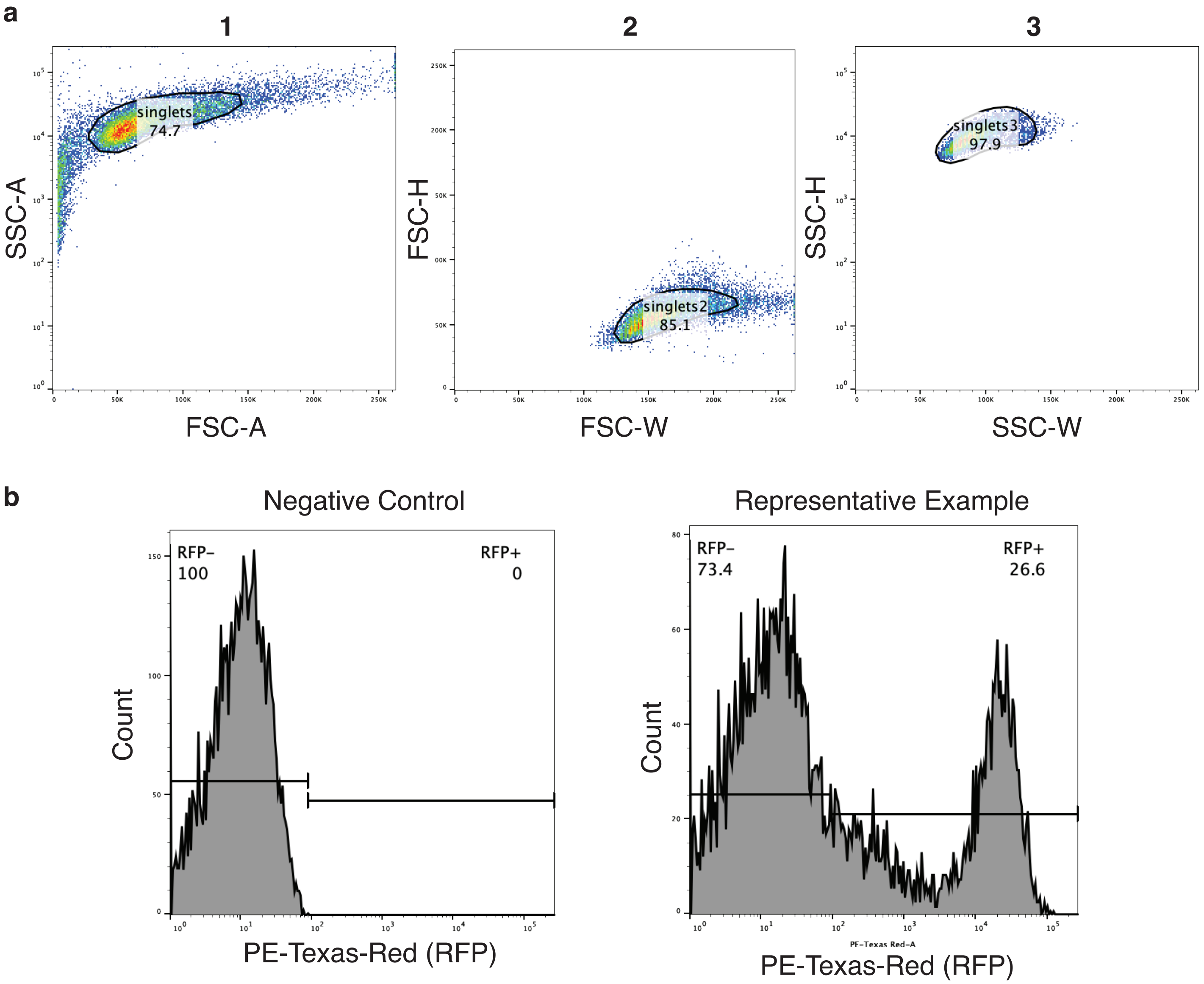

Supplemental Figure 1. Flow gating strategy for competition assay analysis. **a)** Gating strategy for the isolation of single cells. Numbering (1-3) represents the ordering of subpopulations. Population 3 ("singlets3") is final population analyzed for RFP positive cell fraction. **b)** RFP positive cell fraction gating scheme. The untransduced negative control population (left) is used to set the threshold for RFP positivity. A representative example of a non-control population is shown at right.

Supplemental Protocol 1

PE Sensor CRISPR Library Cloning

Workflow:

Amplification → Insert preparation → Ligation → Electroporation

I - Amplification

Reagents needed:

- 2X NEBNext Master Mix (#M0541L) (PCR hood room -20C)
- Oligo pool at 1ng/uL (diluted from reconstituted OLS library; see below)
- Sensor_F and Sensor_R primers:

Sensor_F	Sequence	Sensor_R	Sequence
F	CATAGCGTACACGTCTCACACCG	R	GTGCCGTTGACGACCGGATCTAGAATTC

Reconstitution of lyophilized OLS library:

1. Resuspend the pellet in 100 uL of TE Buffer pH 8.0 or QIAGEN EB Buffer.
2. Incubate at RT for ~1hr and periodically vortex to ensure complete resuspension (or put in a shaking block at RT).
3. Nanodrop and aliquot (typically n=10 aliquots per OLS library) and store these at -20 C.
4. If cloning libraries right away, prepare *fresh* serial dilutions until getting a diluted stock at 1 ng/uL (see below).

Protocol:

- We typically perform n=4 PCR reactions per pool of ~1000 gRNAs (an excess, but allows for plenty of backup insert for subsequent sub-cloning of libraries into different destination vectors, if desired, or to repeat any that fail QC).
    - In this case, performed 32 parallel PCR rxns (~n=1 PCR per pool of ~1000 pegRNAs)
- All PCR reactions should be set up in a PCR hood following standard procedures.
- Always include a water-only control for every primer set to assess non-specific amplification and/or contamination.

1. Reaction conditions (50uL reactions): MAKE A MASTERMIX

- 1 uL of oligo pool* (at 1 ng/uL)
- 1.5 uL of Fwd primer (10 uM stock)
- 1.5 uL of Rev primer (10 uM stock)
- 25 uL of 2X NEBNext
- 21 uL of water
- *The oligo pool should be added outside of the PCR room, preferably in a bench or room that is not used routinely for cloning gRNAs. Never bring oligos or oligo pools into the PCR room.

2. Cycling conditions

- 98 C x 30s
- 98 C x 30s
- 53 C x 30s
- 72 C x 30s
- [**GO TO STEP 2 x 18 total cycles]** Go to step 2 x 24 cycles (note: the # of cycles can be varied and has been tested and optimized; anywhere from 10-24 cycles is fine; less is usually better to minimize potential over-amplification but I haven't found this to be an issue; in fact, most libraries I've cloned have been done w/ 24 cycles; if doing 10 cycles, I would run 8 rxns per pool)
- 72 C x 5 min
- 4 C forever
- Named "CRISPR library cloning 25 cycles" under the library cloning folder in the Mastercycler 1 in Bay 1.

Results for optimization of TP53 PE sensor library cycle count (18 cycles chosen):

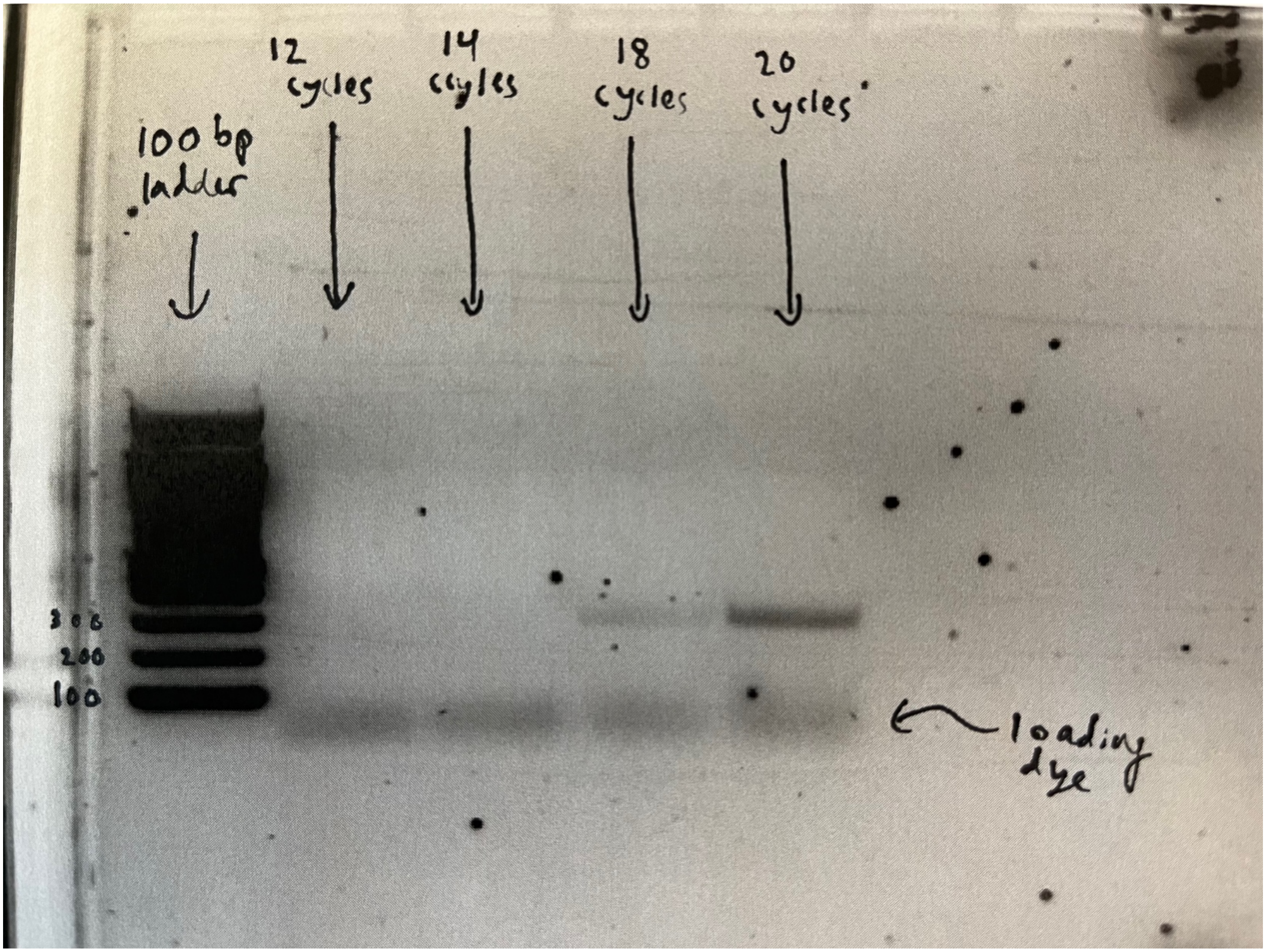

3. PCR purification

1. Pool up to four 50 uL reactions per pool and PCR purify using a single QIAGEN column and standard QIAGEN PCR purification protocol.
2. Add 10 uL of 3M NaOAC pH 5.2 for every 5 volumes of PB used per 1 volume of PCR reaction (e.g. 200 uL pooled rxns need 1 mL of PB + 10 uL NaOAC).
3. Elute in 50 uL of pre-warmed (55 C) EB.
4. Run 5 uL of each purification in a gel (it should look like the gel below).

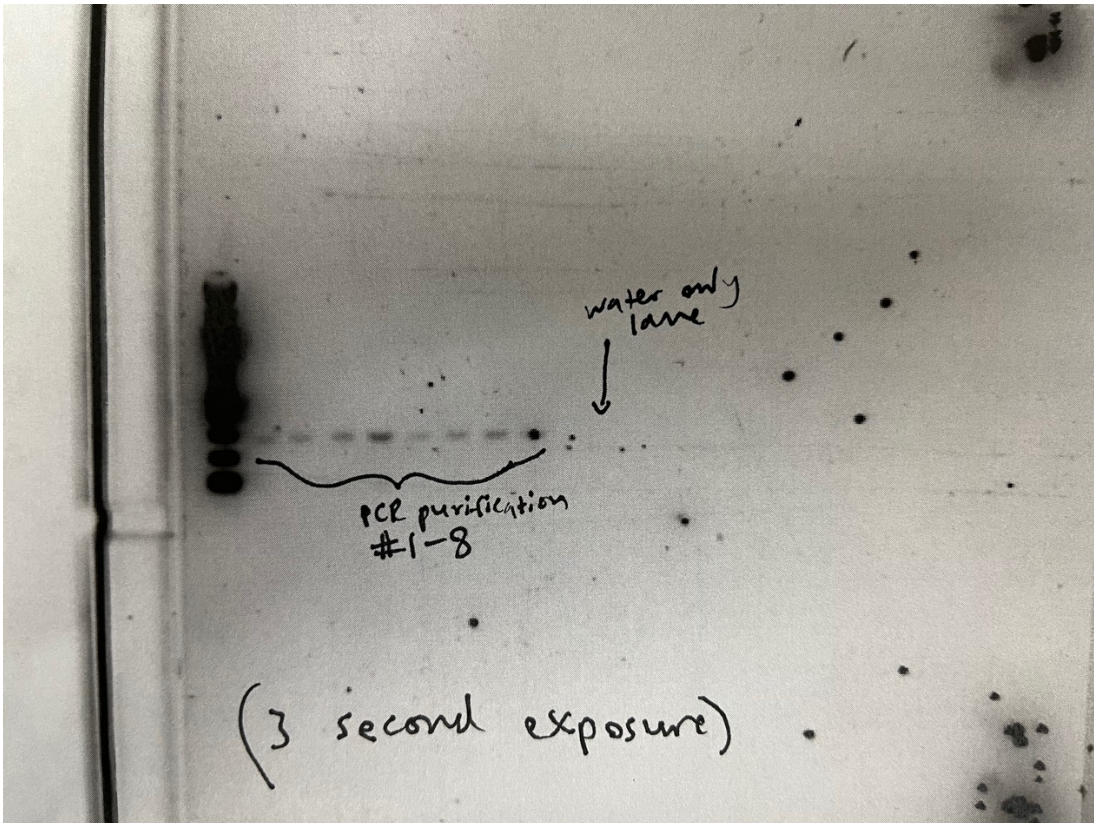

II - Insert preparation

- Insert digestion with Esp3I (NEB) and EcoRI-HF (60 uL reactions)
  - 45 uL purified PCR product **(*8 = 360 uL MM)**
  - 6 uL rCutSmart NEB buffer (10X) **(*8 = 48 uL MM)**
  - 3 uL Esp3I NEB **enzyme (*8 = 24 uL MM)**
  - 3 uL EcoRI-HF NEB Enzyme **(*8 = 24 uL MM)**
  - 3 uL water **(*8 = 24 uL MM)**
  - Digest at 37 C x 4 hrs
- Insert purification
  - Pool up to 4 reactions per pool and PCR purify using a single QIAGEN column.
  - Add 10uL of 3M NaOAC pH 5.2 for every 5 volumes of PB used per 1 volume of PCR reaction.
  - Elute in 30 uL of pre-warmed (55C) EB.

III - Ligation

1. Ligation to cut backbone (20 uL reactions)

    - **Performing n=16 parallel ligations**
    - 6 uL cut and dephosphorylated backbone (at 50 ng/uL)
    - 3 uL insert (at 1 ng/uL)
    - 2 uL T4 ligase buffer (10X)
    - 1 uL T4 ligase (high concentration; #M0202M; do not use low concentration ligase)
    - 8 uL water
    - Incubate at 16 C overnight
2. Precipitation of ligation reactions
    - Pre-spin PhaseLock tubes at max speed for 5 min.
    - Pool up to 4 ligations per pool and complete to 300 uL using water.
    - Add 300 uL of equilibrated Phenol (no Chloroform or Isoamyl alcohol; ensure you pipet from bottom phase).
    - Mix and extract using PhaseLock tubes (spin at max speed for 5min).
    - After spin, take the top 250 uL watery phase, add 25 uL 3M NaOAC pH 5.2, 750 uL ice cooled EtOH and 1.5 uL Pellet paint (Novagen).
    - Mix and store in -20 C (overnight or longer). Can also do -80C for 2hrs.
    - Spin down (13K RPM for 30 min at 4C).
    - Discard supernatant and add ~1 mL of 70% EtOH.
    - Spin down (13K RPM for 5 min at 4C).
    - Repeat for another 70% EtOH wash.
    - Dry and resuspend in pre-warmed EB (55 C) (3 uL of EB per 4 precipitated reactions).

IV - Electroporation

Steps:

1. **Bacterial electroporation**
    A. Dry 10 cm/15 cm LB-Amp / LB-Carb plates at 37 C for at least 6 hrs until completely dry.
    B. Pre-chill cuvettes at -20 C / -80 C throughout the day and store at -20C when ready.
    C. Thaw electrocompetent cells (e.g. Lucigen Endura ElectroCompetent Cells; #60242-2) on ice and aliquot 25uL of bacteria per pre-chilled eppendorf tube per transformation (typically 1 transformation per precipitated ligation reaction, and 1 of these for 1 pool of ~1000 gRNAs).
    D. Add 3uL of precipitated ligation to bacteria.
    E. Incubate on ice for 10min.
    F. Transfer bacteria to pre-chilled cuvette (~28uL) (wipe sides of cuvette w/ kim-wipe before electroporating to remove condensation).

G. Electroporate (manual setting, 2.00 kV, aim for at least 5.2 msec).
H. Rapidly quench w/ ~980uL pre-warmed (37C) SOC or LB.
I. Recover at 37C x 1hr in a bacterial shaker.

2. **Plating**
   A. Dilution plates
      1. Set up serial dilution plates (10E2 - 10E6) by taking 10uL of bacteria and diluting in 990uL SOC/LB (initial 10E2 dilution) and then serially dilute (10uL bacteria + 90uL SOC/LB) until obtaining the 10E6 dilution.
      2. Plate 100uL dilutions into 10cm pre-warmed plates (make sure these are dry).
      3. Spread thoroughly using 4 glass beads per plate until dry.
      4. Incubate inverted at 37C overnight for 16hrs.
      5. Count colonies next day; ideal representation = 10,000X.
      6. SEND FOR SEQUENCING
   B. Library plates
      1. Plate ~240uL of bacteria per 15cm plate (divide in 4 spots in the plate).
      2. Spread thoroughly using 4 glass beads per plate until dry.
      3. Incubate inverted at 37C O/N for 16hrs.
3. **Scraping**
   1. Prepare at least 250mL of fresh LB-Amp per four 15cm plates.
   2. Add 20mL of LB-Amp per 15cm plate.
   3. Scrape using cell lifters.
   4. Transfer to 1L flask.
   5. Repeat steps 1-4.
   6. Complete to 250mL with fresh LB-Amp.
   7. Shake at 37C for at least 2hrs (up to 4hrs).
   8. Spin bacteria and freeze pellets or proceed to large-scale maxipreps.

Supplemental Protocol 2

PCR1/2 protocols
Sam Gould (Sánchez-Rivera Lab)

TP53 PE Sensor Screen Deconvolution

1. Genomic DNA (gDNA) extraction

Samples chosen for extraction:

Sample Name	# Samples	Total Reads Required (MM)
Plasmid Library	1	30
D4_untreated	3	90+
D16_untreated	3	90+
D16_nutlin	3	90+
D25_untreated	3	90+
D25_nutlin	3	90+
D34_untreated	3	90+
D34_nutlin	3	90+
**TOTALS**	**22**	**660+**

Extraction performed using **Qiagen Maxi extraction kit (Genomic-tip 500/G),** following protocol with no modifications.

---

2. PCR1 (Enrichment of sensor & partial adapters)

Name	PCR #	Read Type	Barcode Sequence	Sequence (5' to 3')	Tm (NEB)	Tm (IDT OligoAnalyzer)
Sensor_PCR_F_1	1 (universal)	F	None	CGCTCTTCCGATCTCTAGCGTTCGAGTTAGGAATT	74	64
Sensor_PCR_R_1	1 (universal)	R	None	CTGAACCGCTCTTCCGATCTTTGTGGAAAGGACGAAACACC	78	67.3
Sensor_PCR_F_2	2 (universal)	F	None	AATGATACGGCGACCACCGAGATCTACACCGCTCTTCCGATCTCTAGCGT	83	70.6
**Sensor_PCR_Barcode_X**	2 (custom)	R	NNNNNNNN	CAAGCAGAAGACGGCATACGAGATNNNNNNNNCCTGCTGAACCGCTCTTCCGATCT		

Note: all PCRs are set-up in PCR hood, with template (gDNA) added outside of the hood

PCR mixture:
- 25uL Q5 High-Fidelity 2X Master Mix
- 2.5uL Sensor_PCR_F_1 Primer (10uM)
- 2.5uL Sensor_PCR_R_1 Primer (10uM)

- **10ug** of genomic DNA **(for D19 nutlin gDNA, 10 ug = 6.67 uL)**
- Complete to 50uL w/ H2O. **(if gDNA = 6.67 uL, water = 13.33 uL)**
  - **Remember water-only (no template) control; in this case water = 20 uL**

Optimized PCR program

(1) 98C x 2 minutes
(2) 98C x 10 sec
(3) 60C x 30 sec
(4) 72C x 30 sec
(5) Repeat step 2-4 x 19 cycles (i.e. 20 total cycles)
(6) 72C x 2 min
(7) 4C hold

- Performing 30x PCR reactions with 10 ug of gDNA for each sample (=300 ug/sample); makes sense since cells are hypotriploid, and based on cell counts and DNA extraction numbers
- Normalizing gDNA to 1000 ng/uL (i.e. 1 ug/uL) to make master mixes more simple

- Master mix **for 6 samples (6x 4 strip tubes with 30 PCR rxns/sample = 30*6 = 180 total rxns)**
- (including an excess of 10 rxns)
  - **4.75 mL** =190*25uL Q5 High-Fidelity 2X Master Mix
  - **475 uL** =190*2.5uL Sensor_PCR_F_1 Primer (10uM)
  - **475 uL** =190*2.5uL Sensor_PCR_R_1 Primer (10uM)
  - **1.90 mL** =190*Complete to 50uL w/ H2O. **(10 uL)**
  - **10ug** of genomic DNA **(normalized samples = 10 uL)**
  - **Remember water-only (no template) control with each master mix…**
- For Plasmid library, using 100 ng of plasmid pool as template x7 rxns + 1 water only control
  - (using 10 ng of template failed to produce a strong band)

- Next, 8 PCR purifications performed for each sample (and then pooled)
  - Add 10uL of 3M NaOAC pH 5.2 for every 5 volumes of PB used per 1 volume of PCR reaction.
- Next, a **gel extraction** is performed to extract the PCR1 product
  - **This is a critical step**

---

3. PCR 2 (Illumina adapters and barcodes)

Final PCR mixture:

- 25uL Q5 High-Fidelity 2X Master Mix
- 2.5uL Sensor_PCR_F_2 Primer (10uM)
- 2.5uL **Sensor_PCR_Barcode_X** Primer (10uM) [varies by sample]
- **10 ng** of PCR1 template DNA
- Complete to 50uL w/ H2O.

Optimized PCR program

(1) 98C x 2 minutes
(2) 98C x 10 sec
(3) 67C x 30 sec
(4) 72C x 30 sec

(5) **Repeat step 2-4 x 9 cycles (i.e. 10 total cycles)**
(6) 72C x 2 min
(7) 4C hold

Name	Sample	Barcode Sequence	Sequence (5' to 3')
Sensor_PCR_Barcode_1	Plasmid Library	CGGTTCAA	CAAGCAGAAGACGGCATACGAGATCGGTTCAACCTGCTGAACCGCTCTTCCGATCT
Sensor_PCR_Barcode_2	D4_REP1	GCTGGATT	CAAGCAGAAGACGGCATACGAGATGCTGGATTCCTGCTGAACCGCTCTTCCGATCT
Sensor_PCR_Barcode_3	D4_REP2	TAACTCGG	CAAGCAGAAGACGGCATACGAGATTAACTCGGCCTGCTGAACCGCTCTTCCGATCT
Sensor_PCR_Barcode_4	D4_REP3	TAACAGTT	CAAGCAGAAGACGGCATACGAGATTAACAGTTCCTGCTGAACCGCTCTTCCGATCT
Sensor_PCR_Barcode_5	D16_REP1	ATACTCAA	CAAGCAGAAGACGGCATACGAGATATACTCAACCTGCTGAACCGCTCTTCCGATCT
Sensor_PCR_Barcode_6	D16_REP2	GCTGAGAA	CAAGCAGAAGACGGCATACGAGATGCTGAGAACCTGCTGAACCGCTCTTCCGATCT
Sensor_PCR_Barcode_7	D16_REP3	ATTGGAGG	CAAGCAGAAGACGGCATACGAGATATTGGAGGCCTGCTGAACCGCTCTTCCGATCT
Sensor_PCR_Barcode_8	D16_Nut_REP1	TAGTCTAA	CAAGCAGAAGACGGCATACGAGATTAGTCTAACCTGCTGAACCGCTCTTCCGATCT
Sensor_PCR_Barcode_9	D16_Nut_REP2	CGGTGACC	CAAGCAGAAGACGGCATACGAGATCGGTGACCCCTGCTGAACCGCTCTTCCGATCT
Sensor_PCR_Barcode_10	D16_Nut_REP3	TACAGAGG	CAAGCAGAAGACGGCATACGAGATTACAGAGGCCTGCTGAACCGCTCTTCCGATCT
Sensor_PCR_Barcode_11	D25_REP1	ATTGTCAA	CAAGCAGAAGACGGCATACGAGATATTGTCAACCTGCTGAACCGCTCTTCCGATCT
Sensor_PCR_Barcode_12	D25_REP2	TATGTCTT	CAAGCAGAAGACGGCATACGAGATTATGTCTTCCTGCTGAACCGCTCTTCCGATCT
Sensor_PCR_Barcode_13	D25_REP3	ATTGGATT	CAAGCAGAAGACGGCATACGAGATATTGGATTCCTGCTGAACCGCTCTTCCGATCT
Sensor_PCR_Barcode_14	D25_Nut_REP1	ATACTCGG	CAAGCAGAAGACGGCATACGAGATATACTCGGCCTGCTGAACCGCTCTTCCGATCT
Sensor_PCR_Barcode_15	D25_Nut_REP2	TATGAGAA	CAAGCAGAAGACGGCATACGAGATTATGAGAACCTGCTGAACCGCTCTTCCGATCT
Sensor_PCR_Barcode_16	D25_Nut_REP3	TAACTCAA	CAAGCAGAAGACGGCATACGAGATTAACTCAACCTGCTGAACCGCTCTTCCGATCT
Sensor_PCR_Barcode_17	D34_REP1	CGTGAGCC	CAAGCAGAAGACGGCATACGAGATCGTGAGCCCCTGCTGAACCGCTCTTCCGATCT
Sensor_PCR_Barcode_18	D34_REP2	ATCAGAGG	CAAGCAGAAGACGGCATACGAGATATCAGAGGCCTGCTGAACCGCTCTTCCGATCT
Sensor_PCR_Barcode_19	D34_REP3	TATGGAGG	CAAGCAGAAGACGGCATACGAGATTATGGAGGCCTGCTGAACCGCTCTTCCGATCT
Sensor_PCR_Barcode_20	D34_Nut_REP1	GCGTTCAA	CAAGCAGAAGACGGCATACGAGATGCGTTCAACCTGCTGAACCGCTCTTCCGATCT
Sensor_PCR_Barcode_21	D34_Nut_REP2	CGCAAGAA	CAAGCAGAAGACGGCATACGAGATCGCAAGAACCTGCTGAACCGCTCTTCCGATCT
Sensor_PCR_Barcode_22	D34_Nut_REP3	CGACAGCC	CAAGCAGAAGACGGCATACGAGATCGACAGCCCCTGCTGAACCGCTCTTCCGATCT

- Next, PCR purifications performed for each sample (pooling together all 4 reactions)
  - Add 10uL of 3M NaOAC pH 5.2 for every 5 volumes of PB used per 1 volume of PCR reaction.
- Next, a **gel extraction** is performed to extract the PCR2 product
  - **This is a critical step**

4. Custom sequencing approach for Illumina NovaSeq S1 200 (NovaSeq 6000)

a

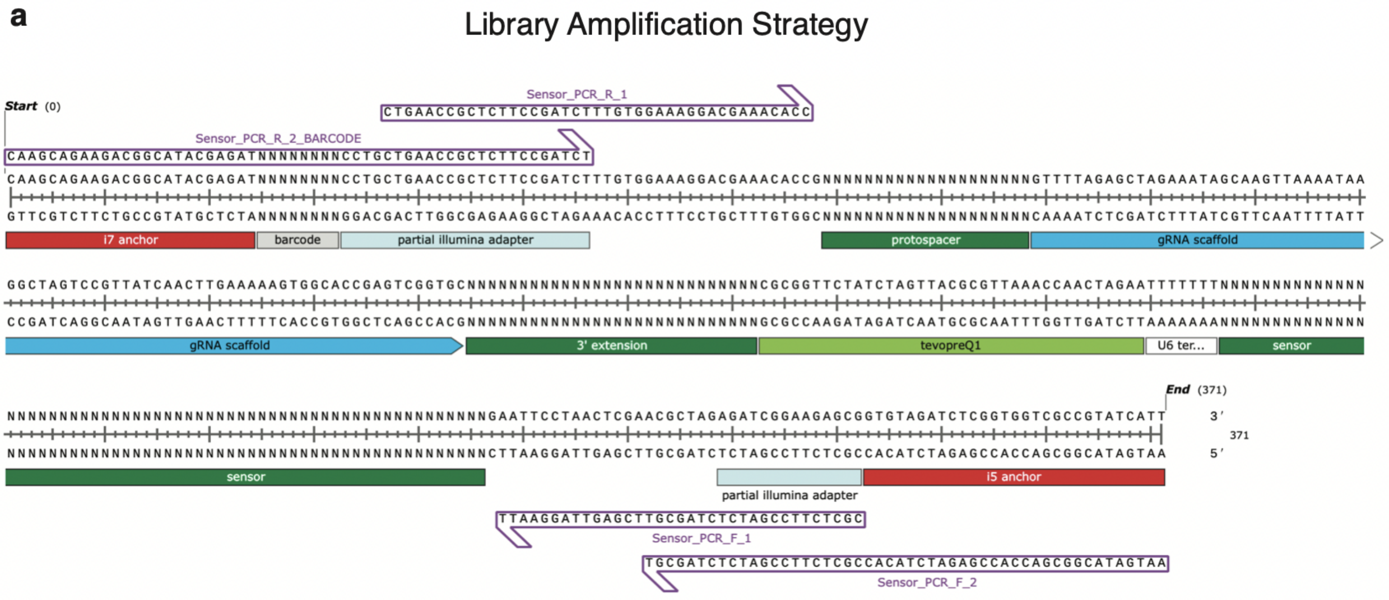

b

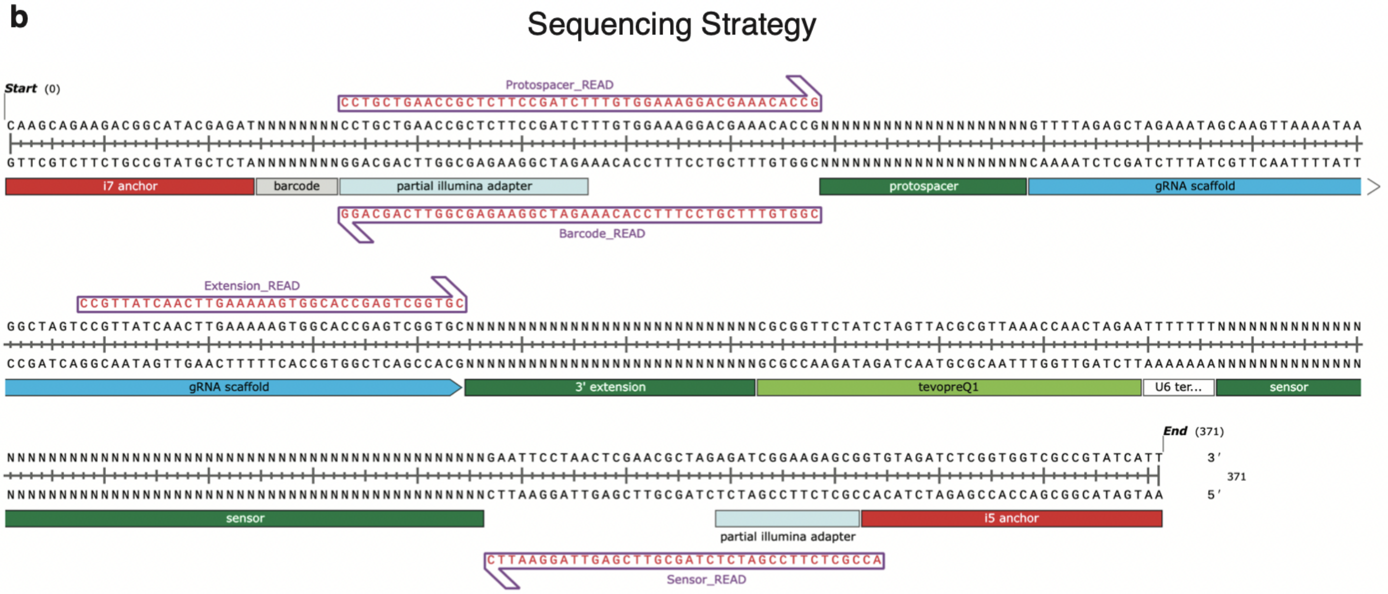

a) Library amplification strategy (PCR1/2)
b) Custom sequencing strategy showing placement of sequencing primers

Singular G4 sequencing of endogenous *TP53* (Figure 4)

1. **PCR1 recipe & protocol (Performed in PCR Hood):**

Perform 12 PCR1 reactions per amplicon with 5 ug of gDNA per reaction:

- **PCR recipe:**
    - 3 sets of Master mix:
        - 25 uL Q5 High-Fidelity 2X Master Mix
        - 15 uL water
        - 2.5 uL F primer (10 uM)
        - 2.5 uL R primer (10 uM)
        - aliquot 45 uL/tube
    - **Add 5 uL = 5 ug gDNA per reaction**
    - **INCLUDE 1 Water only control/master mix**
- **PCR protocol:**
    (1) 98C x 2 minutes
    (2) 98C x 10 sec
    (3) **55C** x 30 sec
    (4) 72C x 30 sec
    (5) Repeat step 2-4 x 19 cycles (i.e. 20 total cycles)
    (6) 72C x 2 minutes
    (7) 4C hold

Singular G4 PCR1 Primers

Sample	Primer Pair	Reaction #
D4-REP1	PCR1_6F, R	1
D4-REP2	PCR1_7F, R	2
D4-REP3	PCR1_10F, R	3
D34-REP1	PCR1_6F, R	4
D34-REP2	PCR1_7F, R	5
D34-REP3	PCR1_10F, R	6
D34-Nutlin-REP1	PCR1_6F, R	7
D34-Nutlin-REP2	PCR1_7F, R	8
D34-Nutlin-REP3	PCR1_10F, R	9
A549 PE-Max WT	PCR1_6F, R	10
A549 PE-Max WT	PCR1_7F, R	11
A549 PE-Max WT	PCR1_10F, R	12

After PCR1:

- Pool 4 PCR1 reactions and PCR purify using a single QIAGEN column.
- Add 10uL of 3M NaOAC pH 5.2 for every 5 volumes of PB used per 1 volume of PCR reaction.
- Elute in 50uL of pre-warmed (55C) EB and pool common samples.
- **Then, gel extract each sample for subsequent PCR2.**

Performing the same PCR1 process for A549 PE-Max Parental (WT) gDNA. However, given the limited amount of gDNA, only performing 3x reactions/amplicon.

---

2. PCR2 Recipe (Performed in PCR Hood)

Sample	Barcoded Primer Pair	Reaction #	Index 1 BC	Index 2 BC
D4-REP1	PCR2_1F, R	1	CGGTTCAA	ATCAGATT
D4-REP2	PCR2_2F, R	2	GCTGGATT	TAGTGATT
D4-REP3	PCR2_3F, R	3	TAACTCGG	CGGTTCGG
D34-REP1	PCR2_4F, R	4	TAACAGTT	TATGGACC
D34-REP2	PCR2_5F, R	5	ATACTCAA	GCCAAGTT
D34-REP3	PCR2_6F, R	6	GCTGAGAA	CGCAGACC
D34-Nutlin-REP1	PCR2_7F, R	7	ATTGGAGG	CGACCTCC
D34-Nutlin-REP2	PCR2_8F, R	8	TAGTCTAA	GCCACTGG
D34-Nutlin-REP3	PCR2_9F, R	9	CGGTGACC	GCGTAGTT
A549 PE-Max WT	PCR2_10F, R	10	TACAGAGG	CGCAAGTT
A549 PE-Max WT	PCR2_11F, R	11	ATTGTCAA	CGACAGTT
A549 PE-Max WT	PCR2_12F, R	12	TATGTCTT	TAGTAGCC

Perform 4 PCR2 reactions per amplicon with 30 ng of PCR1 product as template:

- **PCR recipe:**
  - 3 sets of Master mix:
    - 25 uL Q5 High-Fidelity 2X Master Mix
    - 17 uL water
    - 1 uL F primer (10 uM)
    - 1 uL R primer (10 uM)
    - aliquot 44 uL/tube
  - In each tube, place 30 ng = 6 uL of PCR1 gel purified
  - **INCLUDE 1 Water only control/master mix**

- **PCR protocol:**
    - 98C x 2 minutes
    - 98C x 10 sec
    - **60C** x 30 sec
    - 72C x 30 sec
    - **Repeat step 2-4 x 9 cycles (i.e. 10 total cycles)**
    - 72C x 2 minutes
    - 4C hold

After PCR2:

- Pool 4 PCR1 reactions and PCR purify using a single QIAGEN column.
- Add 10uL of 3M NaOAC pH 5.2 for every 5 volumes of PB used per 1 volume of PCR reaction.
- Elute in 50uL of pre-warmed (55C) EB and pool common samples.
- **Then, gel extract each sample.**

Singular G4 sequencing of endogenous *TP53* in pure cell lines generated for competition assays (Figure 5)

- The same PCR1/2 protocol was used as above.
- However, the number of PCR1 reactions was adjusted according to the amount of gDNA extracted from each pure cell line.
- The PCR2 primers, and associated barcodes, used for each sample are listed in Supplemental Table 2.

Supplemental Protocol 3

pegRNA Golden Gate Assembly

Workflow:

1. Top/bottom oligo design → (synthetic Golden gate assembly in snapgene) → phosphorylation/annealing of oligos → oligo dilution 1:100 → Golden Gate reaction → transformation/plating

This is a general schematic, though with BsaI, rather than BsmBI as the Golden Gate cutter (thus overhangs aren't accurate):

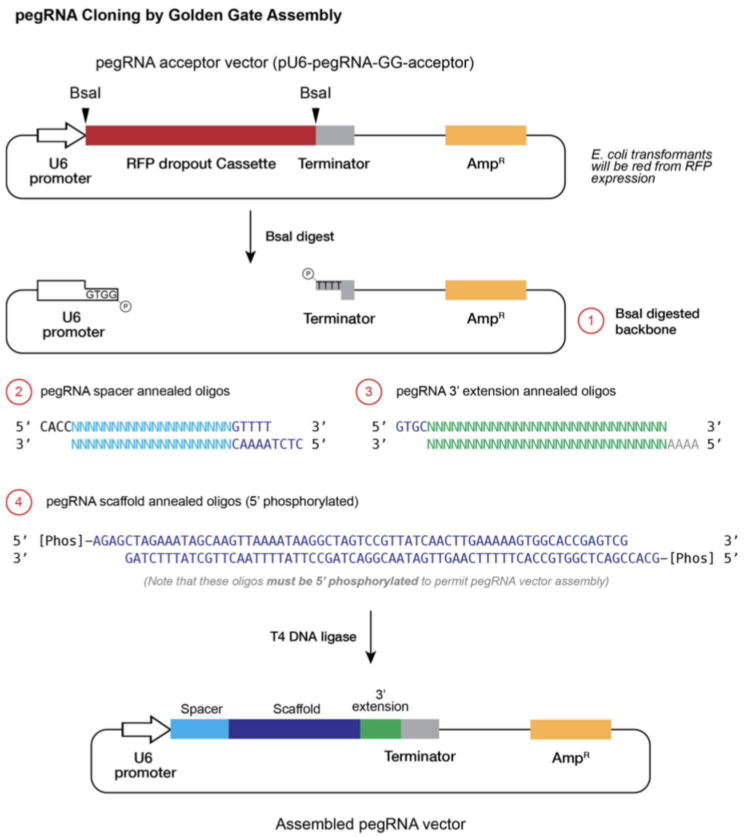

Oligo Design:

Protospacer top/bottom: (5' → 3' and 3' → 5'): **(Make sure protospacer starts with G)**

CACC(G)NNNNNNNNNNNNNNNNNNNNGTTTT

(C)NNNNNNNNNNNNNNNNNNNNCAAAATCTC

Scaffold top/bottom: (5' → 3' and 3' → 5'):

AGAGCTAGAAATAGCAAGTTAAAATAAGGCTAGTCCGTTATCAACTTGAAAAAGTGGCACCGAGTCG

GATCTT TATCGTTCAATTTTATTCCGATCAGGCAATAGTTGAACT TTTTCACCGTGGCTCAGCCACG

3’ Extension top/bottom: (5’ → 3’ and 3’ → 5’):

GTGCNNNNNNNNNNNNNNNNNNNNCGCGGTTCTATCTAGTTACGCGTTAAACCAACTAGAA
NNNNNNNNNNNNNNNNNNNNGCGCCAAGATAGATCAATGCGCAATTTGGTTGATCTTAAAA

Modified version with tevopreQ1 included in backbone:

GTGCNNNNNNNNNNNNNNNNNNNN
NNNNNNNNNNNNNNNNNNNNGCGC

Key: Overhang, Scaffold Region, tevopreQ1 motif

Materials needed:

Materials/Equipment	Location	☐
Top/bottom oligos @ 100 uM	N/A	
T4 DNA Ligase	Bay 2	☐
T4 PNK	-20C (near Bay 8)	☐
10X T4 DNA Ligase Buffer	-20C (near Bay 8)	☐
Nuclease-free (NF) water	Bay 1, 2	☐
NEB Golden Gate Enzyme Mix **(BsmBI)**	-20C (near Bay 8)	☐
UPEmS or UPEmS_tevo (or other vector) @75 ng/uL	-20C	☐

Steps:

1. Run annealing and phosphorylation reactions for oligos

a. There are 3 oligo pairs: (1) protospacer top/bottom, (2) scaffold top/bottom, (3) 3’ extension top/bottom. These oligos should be **generated with the appropriate overhangs** according to the map above. ***Performing an in silico Golden Gate reaction in SnapGene is HIGHLY recommended before proceeding.***

b. Phosphorylate and anneal the **scaffold** oligos. Alternatively, we generally keep a supply of phosphorylated scaffold in stock that can be re-used:

**Component**	**1x**
**Oligo 1 (100 µM)**	**1uL**
**Oligo 2 (100 µM)**	**1uL**

10x T4 DNA ligase buffer	1uL
H_2O	6.5uL
T4 PNK	0.5uL
Run annealing program	

c. Anneal (but do not phosphorylate) the **protospacer and 3' extension oligos** (same reaction as above, but with T4 DNA Ligase instead of T4 PNK):

Component	1x
Oligo 1 (100 µM)	1uL
Oligo 2 (100 µM)	1uL
10x T4 DNA ligase buffer	1uL
H_2O	6.5uL
T4 DNA Ligase	0.5uL
Run annealing program	

Annealing program:

37˚ C	30 mins
95˚ C	5 mins
25˚C FINAL	**Ramp down to 25˚C at 5˚C/min** (i.e. 0.083˚C/sec; 0.1˚C/sec also seems to work fine)

2. Dilute phosphorylated and annealed oligos 1:100 in nuclease-free water.

a. Keep products on ice and store at -20˚C.

3. Perform Golden Gate reaction

Component	1x	12x	5x
UPEmS Vector or other (75 ng/µL)	1.5µL	18	7.5
Protospacer (100nM)	1µL	12	5
scaffold (100nM)	1µL	12	5
3' ext (100nM)	1µL	12µL	5µL
T4 DNA Ligase Buffer (10x)	2uL	24	10
NEB Golden Gate Enzyme Mix	1uL	12	5
H_2O	12.5µL	150	62.5
TOTAL	20uL	240uL	100 uL
(42C, 1min → 16C, 1min) x 60 repeats → 60C, 5min			
Transform and plate the entire 20 uL reaction			

4. Transformation reaction copied for convenience:

Materials needed:

Materials/Equipment	Location	☑
Ice	Hallway (near TC)	☐
Competent cells (very fragile!)	-80 C	☐
Plasmid	-20 C (near Bay 8)	☐
Block for heat shock @ 42 C	Bay 1 in Lab	☐
SOC/LB	Hallway (near the lab entrance)	☐
Petri dishes	Hallway (near the lab entrance)	☐
Glass beads	Bay 2 and 3 in Lab	☐

Steps:

1. Always thaw competent cells on ice as they are quite fragile.
2. Check the tube. Are competent cells at the bottom of the tube? If not, give it the "manual centrifuge" treatment by giving the tube a quick outward whip to bring them to the bottom of the tube.

3. Add **20 uL of the golden gate reaction product** to a vial of competent cells and gently flick the tube. Ensure bacteria are at the bottom of the tube.
4. Incubate on ice for 5-10 min
5. Heat shock at 42 C for 30-45 seconds.
6. Incubate on ice for 3min.
7. Add 250uL SOC or LB-only media. At this point, the bacteria can sit at room temperature for up to 1hr.
8. Plate or inoculate plasmid prepping cultures.
9. Plate 50-100uL bacteria and thoroughly spread with glass beads.
10. Incubate inverted plates at 37C overnight.
