# Supplementary material for: High-throughput evaluation of genetic variants with prime editing sensor libraries
Source: Nat Biotechnol. 2024 Mar 12;43(10):1648–62. doi: 10.1038/s41587-024-02172-9 (PMC12520993; doi:10.1038/s41587-024-02172-9)
Supplement: Supplementary file 2 — Reporting Summary [file 41587_2024_2172_MOESM2_ESM.pdf]

nature portfolio

Corresponding author(s): Samuel I. Gould, Francisco J. Sanchez Rivera

Last updated by author(s): Feb 6, 2024

Reporting Summary

Nature Portfolio wishes to improve the reproducibility of the work that we publish. This form provides structure for consistency and transparency in reporting. For further information on Nature Portfolio policies, see our Editorial Policies and the Editorial Policy Checklist.

Statistics

For all statistical analyses, confirm that the following items are present in the figure legend, table legend, main text, or Methods section.

n/a	Confirmed	
☐	☒	The exact sample size (*n*) for each experimental group/condition, given as a discrete number and unit of measurement
☐	☒	A statement on whether measurements were taken from distinct samples or whether the same sample was measured repeatedly
☐	☒	The statistical test(s) used AND whether they are one- or two-sided<br>*Only common tests should be described solely by name; describe more complex techniques in the Methods section.*
☐	☒	A description of all covariates tested
☐	☒	A description of any assumptions or corrections, such as tests of normality and adjustment for multiple comparisons
☐	☒	A full description of the statistical parameters including central tendency (e.g. means) or other basic estimates (e.g. regression coefficient) AND variation (e.g. standard deviation) or associated estimates of uncertainty (e.g. confidence intervals)
☐	☒	For null hypothesis testing, the test statistic (e.g. *F*, *t*, *r*) with confidence intervals, effect sizes, degrees of freedom and *P* value noted<br>*Give P values as exact values whenever suitable.*
☒	☐	For Bayesian analysis, information on the choice of priors and Markov chain Monte Carlo settings
☒	☐	For hierarchical and complex designs, identification of the appropriate level for tests and full reporting of outcomes
☐	☒	Estimates of effect sizes (e.g. Cohen's *d*, Pearson's *r*), indicating how they were calculated

Our web collection on statistics for biologists contains articles on many of the points above.

Software and code

Policy information about availability of computer code

Data collection	BD FACSDiva v9.0 software was used to collect flow cytometry data.
Data analysis	All custom code for analysis of sequencing and other data, as well as Jupyter notebooks for generating each figure that appears in the paper, is provided at the following Github repository: https://github.com/samgould2/p53-prime-editing-sensor. Additionally, MAGeCK 0.5.9 was used for pegRNA count normalization and LFC quantification and FlowJo 10.9.0 was used to analyze flow cytometry data. Fastq-join 1.3.1 was used to join paired-end sequencing reads. Crispresso2 was used to quantify editing. Further documentation and installation instructions for PEGG are available at pegg.readthedocs.io.

For manuscripts utilizing custom algorithms or software that are central to the research but not yet described in published literature, software must be made available to editors and reviewers. We strongly encourage code deposition in a community repository (e.g. GitHub). See the Nature Portfolio guidelines for submitting code & software for further information.

Data

Policy information about availability of data

All manuscripts must include a data availability statement. This statement should provide the following information, where applicable:

- Accession codes, unique identifiers, or web links for publicly available datasets
- A description of any restrictions on data availability
- For clinical datasets or third party data, please ensure that the statement adheres to our policy

Raw sequencing data from the screen is deposited in the SRA under accession PRJNA1014453. MSK-IMPACT clinical sequencing data was accessed from the cBioPortal (https://www.cbioportal.org/). Data for the Giacomelli et al. (2018) cDNA comparison was accessed from Supplementary Table 3 in the corresponding manuscript (https://doi.org/10.1038/s41588-018-0204-y).

Human research participants

Policy information about studies involving human research participants and Sex and Gender in Research.

Reporting on sex and gender	Not applicable.
Population characteristics	Not applicable.
Recruitment	Not applicable.
Ethics oversight	Not applicable.

Note that full information on the approval of the study protocol must also be provided in the manuscript.

Field-specific reporting

Please select the one below that is the best fit for your research. If you are not sure, read the appropriate sections before making your selection.

☒ Life sciences ☐ Behavioural & social sciences ☐ Ecological, evolutionary & environmental sciences

For a reference copy of the document with all sections, see nature.com/documents/nr-reporting-summary-flat.pdf

Life sciences study design

All studies must disclose on these points even when the disclosure is negative.

Sample size	The sample size of the cell population of each replicate in the screen, as well as minimum cell passage numbers, were determined with the goal of maintaining 1000x representation of the pegRNA-sensor library at every step of the screen. Initial transductions of each replicate included 110 million cells at low MOI, and subsequent passages maintained at least 30 million cells (>1000X representation) for each replicate. Maintaining >1000X representation was chosen to be a sufficient sample size because this represents the standard in the field among high throughput screens.
Data exclusions	We excluded pegRNA-sensor cassettes with fewer than 10 reads from subsequent analysis of pegRNA editing efficiency and filtered log-fold change (LFC) because of the low confidence in editing activity provided by sensors with low sample sizes. We also excluded pegRNAs with fewer than 10 control reads (day 4) from enrichment analysis because of the potential for spuriously enriching pegRNAs in these cases.
Replication	The prime editing sensor screen was performed in triplicate, with three separate transductions performed. All fluorescence competition assays were also performed in triplicate for each condition. In general, all replicates behaved in highly similar manner.
Randomization	During the prime editing sensor screen, each replicate was split into two treatment arms (untreated and Nutlin-treated) by taking an equal, randomly sampled subset of re-suspended cells from the same replicate. In competition assays, cell populations were randomly split into different treatment arms (untreated and Nutlin-treated).
Blinding	Readouts of editing activity and cell growth were unbiased measurements obtained in a blind fashion using sample naming schemes that avoided bias from knowing the identity of the sample.

Reporting for specific materials, systems and methods

We require information from authors about some types of materials, experimental systems and methods used in many studies. Here, indicate whether each material, system or method listed is relevant to your study. If you are not sure if a list item applies to your research, read the appropriate section before selecting a response.

Materials & experimental systems

n/a	Involved in the study
☒	☐ Antibodies
☐	☒ Eukaryotic cell lines
☒	☐ Palaeontology and archaeology
☒	☐ Animals and other organisms
☒	☐ Clinical data
☒	☐ Dual use research of concern

Methods

n/a	Involved in the study
☒	☐ ChIP-seq
☐	☒ Flow cytometry
☒	☐ MRI-based neuroimaging

Eukaryotic cell lines

Policy information about cell lines and Sex and Gender in Research

Cell line source(s)	A549 cells were originally derived from a white, 58-year old male with lung cancer. They were provided to us through the Koch Institute ES Cell and Transgenics Core Facility, who obtained them from the ATCC and subsequently authenticated and mycoplasma tested. HEK293T cells, used for lentivirus production, were also supplied by the Koch Institute ES Cell and Transgenics Core Facility, following the same procedures.
Authentication	Cell lines were authenticated with STR profiling (see statement above).
Mycoplasma contamination	All cell lines tested negative for mycoplasma contamination.
Commonly misidentified lines (See ICLAC register)	No commonly misidentified cell lines were used in this study.

Flow Cytometry

Plots

Confirm that:

☒ The axis labels state the marker and fluorochrome used (e.g. CD4-FITC).

☒ The axis scales are clearly visible. Include numbers along axes only for bottom left plot of group (a 'group' is an analysis of identical markers).

☒ All plots are contour plots with outliers or pseudocolor plots.

☒ A numerical value for number of cells or percentage (with statistics) is provided.

Methodology

Sample preparation	A549-PEmax cells were trypsinized and resuspended, and then 200 uL of each sample, including uncolored controls for gating purposes, were placed in a U-bottom 96-well plate.
Instrument	BD FACSCelesta Cell Analyzer
Software	FlowJo 10.9.0 was used to analyze flow cytometry data.
Cell population abundance	We applied a stringent threshold of ≥ 500 quantifiable events (i.e. single cells) because we found that samples with ≤ 500 quantifiable events, which were typically observed in cells treated with Nutlin-3 that underwent cellular senescence and/or apoptosis, were insufficient to accurately calculate the RFP positive cell fraction. In these cases, we assumed that the RFP positive cell fraction was unchanged from the previous time-point, akin to a standard 3T3/proliferation assay.
Gating strategy	FSC/SSC gates were used to define single cell populations using WT, uncolored A549-PEmax cells. Uncolored A549-PEmax cells were also used to establish an RFP-negative threshold/gate.

☒ Tick this box to confirm that a figure exemplifying the gating strategy is provided in the Supplementary Information.
